# Review of "Bioterrorism: Mathematical modeling applications in homeland security" by H. T. Banks and C. Castillo-Chavez

**DOI:** 10.1186/1475-925X-4-69

**Published:** 2005-12-22

**Authors:** Ming Jiang

**Affiliations:** 1 LMAM, School of Mathematical Sciences, Peking University, Beijing 100871, China

According to the ISCID Encyclopedia of Science and Philosophy, "*Bioterrorism is a type of terrorism that employs the use of biological agents to achieve its goals*" [1]. This has been a hot topic in the United States since the mysterious anthrax letters of fall 2001 [2]. Mathematical models of viral transmission and control are important tools for assessing the threat posed by deliberate release of the biological agents such as smallpox, influenza and foot and mouth disease (FMD), *etc*., and the best means of containing an outbreak [3]. Development in this field should provide planning tools to help manage emerging infections of all kinds.

From the preface: "*This volume brings the contributions of a selected group of experts from various fields who have begun to develop models to tackle some of the current challenges raised in biosurveillance, agroterrorism, bioterror response logistics, and assessment of the impact of the deliberate release of biological agents and the social forces that maintain groups of terror*."

This book is engendered from a workshop in 2002 sponsored by the Center for Discrete Mathematics & Theoretical Computer Science (DIMACS) at Rutgers University and the National Science Foundation (NSF). There is an introductory preface about the book and ten chapters presenting mathematical models related to bioterrorism

The 1^st ^chapter, "*Challenges for Discrete Mathematics and Theoretical Computer Science in the Defense against Bioterrorism*", is an overview of the approaches based on the discrete mathematics and theoretical computer science to the bioterrorism issues. It presents an analysis of detailed challenges for discrete mathematics and theoretical computer science and relevant tools. "*These tools, which seem very relevant to epidemiological problems and in particular to the defense against bioterrorism, are not very well known among those working on public health problem*."

The 2^nd ^chapter, "*Worst-Case Scenarios and Epidemics*", is to identify under what conditions random mixing can be used to model worst-case scenarios. It focuses on a preliminary examination of the validity of the assumptions and tries to solve the disagreement in previous studies because of the preassumed population structure and mixing topology.

The 3^rd ^chapter, "*Chemical and Biological Sensing: Modeling and Analysis from the Real World*", introduced techniques to detect minute amount of chemical and biological substances prior to their dissemination on a large scale. The 1^st ^topic is the TNT molecule detection technique based on a continuous flow displacement immunosensor (CFI sensors) and its improvement with a pulsatile flow in a reaction-convection model. The 2^nd ^topic is about cell-base biosensors. The authors describe two new biosensor methods for short data sets that use area measurements to detect subtle change in the signal. The 3^rd ^topic is how to analyze data from a high-dimensional theoretical model and discover external and internal changes in a continuum mechanical system.

The 4^th ^chapter, "*The Distribution of Interpoint Distances*", shows how to use the distribution of the interpoint distance between two randomly selected observations as a summary of a spatial distribution. The method was applied in practice for syndromic surveillance and generated "*a remarkable constancy over time*".

The 5^th ^chapter, "*Epidemiologic Information for Modeling Foot-and-Mouth Disease*", is a survey of FMD including its history, agent, clinical signs, pathogenesis, prevention and control, and available models, *etc*. All the topics are appropriately discussed with a list of 140 references.

The 6^th ^chapter, "*Modeling and Imaging Techniques with Potential for Application in Bioterrorism*", discusses modeling and imaging techniques. "*The first focuses on physiologically based pharmacokinetic (PBPK)-type models and the effects of drugs, toxins, and viruses on tissue, organs, individuals, and populations wherein both intra- and interindividual variability are present when one attempts to determine kinetic rates, susceptibility, efficacy of toxins, antitoxins, etc. in aggregate populations*. " The method was applied to model progression of HIV at the cellular level infection pathway as an example. "*A second effort concerns the use of remote electromagnetic interrogation pulses linked to dielectric properties of materials to carry out macroscopic structural imaging of bulk packages (drugs, explosives, etc.) as well as test for presence and levels of toxic chemical compounds in tissue*."

The 7^th ^chapter, "*Models for the Transmission Dynamics of Fanatic Behaviors*", "*explicitly focuses on the generation of qualitative results that may be useful in the fight against fanaticism, the force behind the acts of terrorism*." However, "*the results in this chapter should not be taken as a prediction of reality*", because the situation is highly complex and there is the issue of data to validate the model.

The 8^th ^chapter, "*An Epidemic Model with Virtual Mass Transportation: The Case of Smallpox in a Large City*", studies release targets that include city subway system or airport hubs. An epidemiological model for the transmission dynamic and control of smallpox with multiple level of mixing is introduced together with simulation results. "*In summary, having enough vaccines stockpiles, while necessary, is not enough. The best policy must include the timely application of mass vaccination*." Chapter 2 addresses the disagreement on this issue.

The 9^th ^chapter, "The *Role of Migration and Contact Distributions in Epidemic Spread*", presents a reaction-diffusion model for epidemic spread with a sedentary and a migrating compartment. The author demonstrated how epidemic models revealing the appropriate epidemiological scenario can be obtained by scaling of parameters.

The 10^th ^chapter, "*Modeling the Spread of Influenza among Cities*", uses a modified susceptible-infected-recovered (SIR) model for the spread of influenza in each city and assumes nonrandom mixing of the population among the cities by the air travel. The model parameters are estimated from the literature. The authors provide a comparison of the model predictions with the CDC data for several representative cities. "*This simple model did remarkably well at predicting the yearly influenza epidemic*."

From the preface: "*Naturally, this volume gives a limited view of the challenges, possibilities, and opportunities that may be tackled with the use of mathematical and computational approaches. Our hope is that the contributions in its 10 chapters may inspire and stimulate research at the interface of homeland security and the mathematical and statistical sciences*. "

Each chapter has its own list of references. There is an index for the entire book. While generally speaking it is in high quality, there are some symbols used without definitions. For example, the symbols *Ab *and *Ag *on p. 58 are not introduced before its first use. Some representations could have been done better. For example, some results are given in tables of numbers, for which graphic presentations should be more illustrative.

From the publisher: "*This volume will be of interest particularly to those specializing in the biological sciences including biophysics, biomedical engineering, and biomathematics, but its applications extend to readers with an interest in control and systems theory, dynamical systems, inverse problems, discrete math, statistics, computational science, and, of course, simulation and modeling*."

Other reviews of this book can found at the MAA Book Review Column and the book review section in Bulletin (New Series) of the American Mathematical Society[4,5]. Readers are recommended to read the recent reviews on the mathematical models and various aspects in bioterrorism [3,6].
